# Guanidine Derivatives Leverage the Antibacterial Performance of Bio-Based Polyamide PA56 Fibres

**DOI:** 10.3390/polym16192707

**Published:** 2024-09-25

**Authors:** Lili Wang, Bobo Zhou, Yuliu Du, Miao Bai, Xiang Xu, Yong Guan, Xiucai Liu

**Affiliations:** 1 School of Materials Science and Engineering, Shanghai Key Laboratory of Advanced Polymeric Materials, East China University of Science and Technology, Shanghai 200237, China; y12212099@mail.ecust.edu.cn (L.W.); y30220749@mail.ecust.edu.cn (B.Z.); xiangxu@ecust.edu.cn (X.X.); 2 Shanghai Cathay Biotech Inc., Ltd., Shanghai 201144, China; duyuliu@cathaybiotech.com (Y.D.); baimiao@cathaybiotech.com (M.B.)

**Keywords:** bio-based PA56, guanidine derivative, antibacterial activities, dyeing

## Abstract

Bacterial damage has significantly impacted humanity, prompting the control of harmful microorganisms and infectious diseases. In this study, antibacterial bio-based PA56 fibres were prepared with high-speed spinning using ethylene-methyl acrylate-glycidyl methacrylate terpolymer (EMA) as the compatibiliser and polypentamethylene guanidine sulphate (PPGS) as the antibacterial agent. The effects of PPGS content on the properties of PA56 draw-textured yarns (DTYs) were investigated. The compatibility between PPGS and PA greatly improved with EMA incorporation. Compared with PA56 fibres, the elongation at break of the sample containing 2.0 wt% EMA and PPGS increased by 25.93%. The inhibition rates of the fibres with 1.0 wt% PPGS against *Escherichia coli* and *Staphylococcus aureus* reached over 99.99%. Samples were easily coloured with dyes, exhibiting good colour fastness, regardless of the EMA content. However, the antibacterial performances of dyed DTYs decreased to varying degrees. the inhibition rates of samples of 0.5wt% addition of PPGS against *E. coli* were reduced from 99.99% to 28.50% and 25.36% after dyeing with Acid Blue 80 and Dispersible Blue 2BLN, respectively. The EMA-modified fibres exhibited the best antibacterial activity after dyeing with neutral gray 2BL. These findings are expected to promote the wider use of biobased PA56 in practical applications that require antibacterial performance and to guide the dyeing process of antimicrobial fibres.

## 1. Introduction

Polyamide (PA), also known as nylon, is the first discovered synthetic fibre [1,2,3]. Most PA fibres in the market are produced from non-renewable fossil fuels, involving production processes that produce significant pollution and carbon emissions [4]. Therefore, environmentally friendly bio-based PA, such as PA56, which is prepared from 1,5-pentane diamine (PDA) through biological conversion, has garnered widespread attention [5,6]. Previous studies noted the good dyeing, softness, and antistatic properties of PA56 spun fibres [7,8,9]. Further, their good moisture absorption provides great opportunities for textiles [10].

Various textile products are used extensively in our daily life [11,12,13]. However, textiles can serve as breeding grounds for pathogens, and harm human health and environmental hygiene. Therefore, the development of antibacterial fibres is valuable. Antibacterial products are typically prepared by adding one or several specific antibacterial agents [14,15,16,17,18]. A series of substituted guanidine has been reported to have antimicrobial properties in the 1930s, thus, guanidine derivatives have been investigated as medicines, crop protection agents, and antiseptics for industrial products, food, and other consumer goods because of their high efficiency, broad antibacterial activity, safety and non-toxicity [19,20,21]. Guanidine derivatives, particularly polyhexamethylene guanidine hydrochloride (PHMG) and polyhexamethylene biguanide (PHMB), are promising antibacterial agents [22,23]. Polypentamethylene guanidine sulphate (PPGS) replaces hexanediamine as the petroleum-based raw material in PHMG with a bio-based glutardiamine, and replaces chloride ions with sulphate ions using anion exchange resins [24]. Consequently, this reduces the metal corrosion due to chloride ions in traditional guanidine oligomers [25,26]. In addition, the development and utilisation of biomass resources can reduce the pressure on non-renewable resources and minimise related environmental problems.

Melt spinning is the most widely used method for producing and modifying PA fibres. Zhang et al. [27] reported the preparation of PHMG and PA56 blend modified antibacterial fibres by melt spinning and characterised their properties. However, the incompatibility of PA56 with PHMG, leaching of PHMG, and the decline of mechanical properties of the fibres limit their application. Ethylene-methyl acrylate-glycidyl methacrylate terpolymer (EMA) is often used as a compatibiliser for modifying polymers [28]. Owing to the presence of epoxy functional groups, EMA also reacts with various chemical groups and has high reactivity. 

Dyeing is an important process in the textile industry, which enhances the product value, satisfies market demands, and promotes industry development [29,30]. Xue et al. [31] reported a PA56 nanofibre membrane (PAM) via electrospinning. Reactive red 141 dye was used to react with the amino group of PAM to obtain a dyed membranes (P–Dye). Subsequently, PHMB was ligated to P–Dye to obtain P–Dye–PHMB. The antibacterial activity of the P–Dye–PHMB membrane against *Pseudomonas putida* and *Escherichia coli* (*E. coli*) were 65±2.1% and 57±2.5%, respectively. However, the antibacterial effect of the modified fibres decreased after dyeing with reactive dye. The guanidine salt used to impart antimicrobial activity to PA textile materials may interfere with fabric dyeing [32], similarly, dyes may affect the antibacterial properties of guanidine salt. Therefore, the choice of dye for the fiber after antibacterial modification is particularly important.

In this study, bio-based PA fibres with good antibacterial properties were prepared by melt-spinning PPGS, PA56, and EMA. This process improved the compatibility of PA56 and PPMG by the EMA ring-opening reaction, reduced the dissolution of the antibacterial agent, and improved the mechanical properties of the fibres. Acid Blue 80, Neutral Grey 2BL, and Dispersed Blue 2BLN were used to dye the PA56 fibres. The effect of PPGS as a cationic guanidine-salt antibacterial agent on the colour uptake and fastness, and antibacterial performance of the PA56 fibres were systematically studied. Compared with previous studies, the modification process described in this work can be continuously reactive, can easily achieve industrialisation, and is more closely related to the dyeing industry. The technical findings are expected to guide the application of antimicrobial technology in developing functional PA-based textiles. In addition, the antibacterial agent PA56 has potential application in various fields such as carpets, luggage interiors, and textiles.

## 2. Materials and Methods

### 2.1. Materials

Guanidine hydrochloride was purchased from Sinopharm Chemical Reagent Co. Ltd.,Shanghai, China. Sulphuric acid was purchased from Sinopharm Group Chemical Reagent Co. Ltd., Shanghai, China. PDA and PA56 (T-1121) master batch were obtained from Cathay Industrial Biotechnology (Shanghai, China). AX8900 EMA (ethylene: methyl acrylate: glycidyl methacrylate = 68:24:8 wt%) was sourced from ARKEMA, Colombes, France. Sodium hydrogen phosphate and monobasic potassium phosphate were purchased from Suzhou Anerdan Fine Chemical Co., Ltd., Suzhou, China. A plate-count agar was purchased from Nantong Feiyu Biological Technology Co., Ltd., Nantong, China. Acid Blue 80 dye was purchased from Hangzhou Huade Chemical Co., Ltd., Hangzhou, China. Neutral grey 2BL dye was purchased from Jiangsu Aosheng Enterprise Development Co., Ltd., Suzhou, China. Disperse Blue 2BLN dye was purchased from Zhejiang Kaisheng Chemical Co., Ltd., Zhejiang, China. *E. coli* (ATCC 8099) and *Staphylococcus aureus* (*S. aureus*, ATCC 6538) were provided by Shanghai Centre for Disease Control and Prevention, Shanghai, China.

### 2.2. Preparation of Functional Antibacterial PA56 Fibres

PPMG was synthesised via the melt polycondensation of PDA and guanidine hydrochloride [33]. Subsequently, ion exchange was performed to replace chloride ions with sulphate ions in a 15 wt% PPMG aqueous solution, and excess water was removed to obtain a pure antibacterial agent, referred to as PPGS [24].The detailed information for this method is presented in Section S1.1 (Supporting Information).

Different ratios of PA56, EMA (0.5–10.0 wt%), and PPGS (0.5–10.0 wt%) were mixed and added to a twin-screw extruder to yield blend chips. Subsequently, the blend chips were dried in a vacuum drum dryer at 105 °C for 8 h to reduce the moisture content to approximately 400 ppm. Antimicrobial PA56 pre-oriented yarn (POY) was obtained by melt spinning using a TITA Machinery spinning machine with a spinneret containing 36 holes, at a take-up speed of 4200 m/min and a spinning temperature of 285 °C. The diameters of the fibres were controlled by adjusting the flow rate of a metering pump.

**Figure 1:**
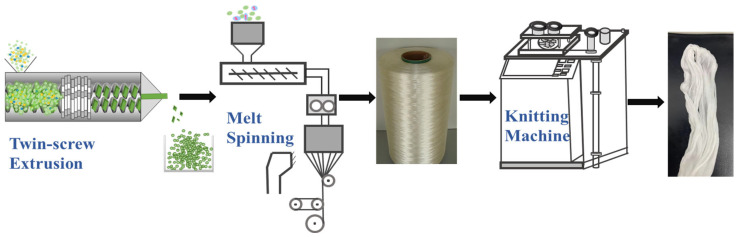
Schematic of PA56 fibres modification process.

PA56 draw-textured yarns (DTYs) were prepared by hot stretching at a ratio of 1.25 times that of the POY using a stretching instrument. To facilitate the subsequent dyeing experiments, the stretched fibres were woven into stockings using a knitting machine (GE598, Wuxi Jiang Kang Textile Mechanical Co. Ltd., Wuxi, China). This process is shown in Figure 1. The sample details are presented in Table 1.

### 2.3. Dyeing of Stockings

The chemical structures of the dyes are listed in Table S1. The stockings were added to a prepared dye solution consisting of Acid Blue 80, Neutral Grey 2BL, and Disperse Blue 2BLN at concentrations of 1.0, 2.0, and 1.0 wt%, respectively (based on the weight of the fabric) using 1:50 material to liquor ratio. The temperature of the dye bath was increased to 98 °C at a heating rate of 1 °C/min and maintained for 30 min. Subsequently, the dyed samples were rinsed with water and air-dried.

### 2.4. Characterization

Fourier transform infrared (FTIR) spectroscopy (Nicolet 5700 Spectrometer, Waltham, MA, USA) was used to analyse the molecular structure of the samples in the wavenumber range of 400–4000 cm^−1^. All samples were prepared using the KBr tablet method.

The section morphologies of samples were examined by scanning electron microscopy (SEM, Hitachi S-4800N, Tokyo, Japan) at the accelerating voltage of 15 kV. All specimens were coated with a thin gold layer to avoid charging, thereby improving the image quality. Energy-dispersive X-ray spectrometry (EDS, Bruker AXS, Karlsruhe, Germany) was employed to analyse the sulphur element distribution on the surface of the modified PA56 fibres.

The thermal stabilities of samples were studied using thermogravimetric analysis (TGA, STA409PC, Netzsch, Germany). All tests were conducted under a nitrogen atmosphere (20 mL/min) with approximately 6 mg samples over a range of 30–600 °C at a heating rate of 10 °C/min.

The tensile properties of PA56 fibres were tested on Automatic Single-Yarn Strength Tester (YG023B-Ⅲ, Changzhou, China) with the stretching rate of 500 mm/min. The clamping lengths of the samples were 500 mm. Each sample was tested 10 times. The average value was calculated and taken in each of the groups.

The inhibition rates against *E. coli* and *S. aureus* were measured using the shaking flask method. The freshly cultured *E. coli* and *S. aureus* were diluted with sterile phosphate-buffered saline (PBS) to 10^5^ CFU/mL. The samples were cut into small pieces before the experiment. After mixing 0.1 mg sample with 10 mL bacterial suspension, the dilutions were shaken at 37 °C at 250 rpm for 24 h. Subsequently, 1 mL of the above bacterial suspension was added to 9 mL PBS, which was labelled as 10^4^. Bacterial suspension 10^3^ and 10^2^ were obtained by the gradient dilution method. Accordingly, 0.1 mL of each bacterial suspension group was inoculated on Luria broth nutrient agar, inverted, and incubated at 37 °C for 24 h. The colonies of bacteria of the blank control and sample were recorded as A and B, respectively. The inhibition rate (R) of the sample was calculated by

(1) $$\%R=\left(\frac{A-B}{A}\times 100\right)$$

The dye concentrations of the bath were measured at the maximum absorption wavelength (λ_max_) before and after dyeing using a UV spectrometer (Lambda 950, Perkin Elmer, Waltham, USA), employing a path length of 1 cm and water as the reference solvent. The dye uptake was calculated using the following Equation:

(2) $$Dye\;uptake=\left(\frac{A_{1}-A_{2}}{A_{1}}\times 100\%\right)$$

where *A*_1_ and *A*_2_ are absorbances of the solutions at *λ*_max_ before and after dyeing, respectively.

The dye uptakes of Acid blue 80 and Neutral Grey 2BL were obtained at the wavelength of 626 and 584 nm, respectively.

The reflectance of the dyed fabrics was measured using a Spectraflash spectrophotometer (Datacolor 650, Lawrenceville, NJ, USA) under illuminant D65 using 10° standard observers. The colour strength (K/S) was calculated from the reflectance value using the following Equation,

(3) $$\frac{K}{S}=\left(\frac{1-R^{2}}{2R}\right)$$

where *K* is the coefficient of absorption, *S* the coefficient of scattering, and *R* the reflectance of the sample at a given wavelength. Each fabric was folded twice to increase the the thicknesses by four folds. The average of three readings was obtained.

The washing fastness was tested using SW-12A tester (Wenzhou Darong Textile Instrument Co., Ltd., Wenzhou, China), in accordance with ISO 105-C10 (2006) standard using 3 g/L standard soap, with drying at 60 °C for 30 min.

The optimised geometry of Disperse Blue 2BLN was obtained by the Gaussian 09 software at the B3LYP/6-31G(d) level. Subsequently, the electrostatic potential at the van der Waals surface defined by Bader (i.e., an electron isodensity surface of 0.001 e/bohr^3^) was calculated using the Multiwfn software.

## 3. Results and Discussion

### 3.1. Sample Synthesis

**Figure 2:**
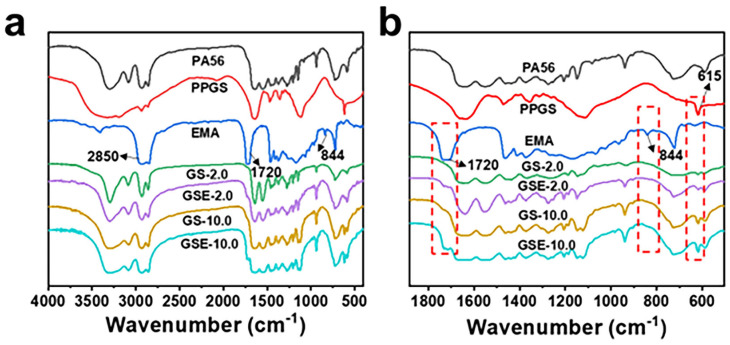
FTIR spectra over the range of (**a**) 4000–400 cm^−1^ and (**b**) 1900–500 cm^−1^.

FTIR spectroscopy was used to investigate the molecular structures of PA56, EMA, and PPGS. As shown in Figure 2, the characteristic peak of the –CH_3_ groups is observed at 2850 cm^−1^. The peak at 1720 cm^−1^ is attributed to the C=O stretching vibration absorption of ester groups that at 844 cm^−1^ is ascribed to the epoxy groups in EMA [34,35].

**Figure 3:**
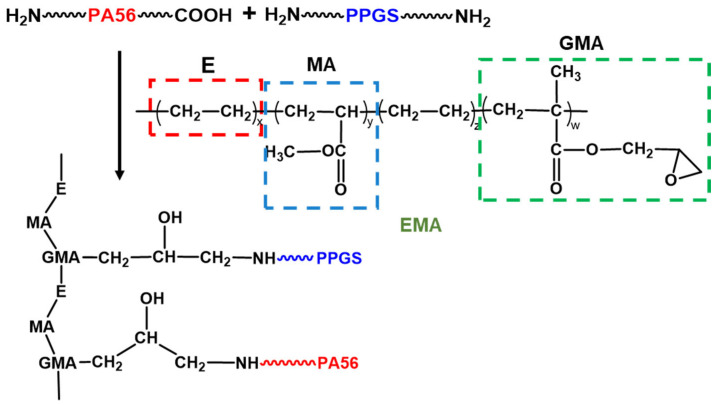
Scheme of preparation of modified PA56 fibres using EMA as the compatibilizer.

The sharp peak at 615 cm^−1^ is attributed to the bending vibrations of –SO_4_^2−^, confirming the presence of PPGS in the modified PA56 fibres. Compared with the spectrum of the blend fibres, different bands are observed in the spectrum of copolymerised fibres (Figure 2b). The peak at 1720 cm^−1^ is attributed to EMA addition. However, the absorption peak of the EMA epoxy groups at 844 cm^−1^ is not observed in the spectrum of GSE-2.0, indicating that the epoxy groups of EMA participated in the chemical reaction [36,37,38]. Even when the dosage of EMA is increased to 10 wt%, epoxy groups are not observed in the FTIR spectrum of GSE-10.0. The FTIR spectroscopy results confirm the successful bonding of EMA and PPGS to the PA56 macromolecules. The in situ compatibilisation mechanism is depicted in Figure 3.

### 3.2. Sample Morphology

**Figure 4:**
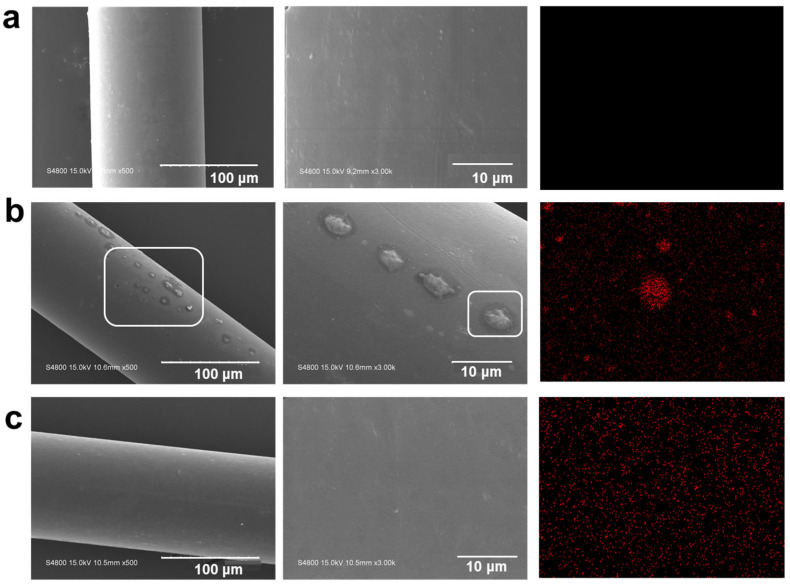
SEM images and sulphur EDS maps of treated PA56 fibres: (**a**) pure PA56, (**b**) GS-2.0, and (**c**) GSE-2.0.

Figure 4 shows the SEM micrographs of the PA56, GS-2.0 and GSE-2.0 surfaces. The sulphur signal, indicated by the red spots in the EDS maps, represents the presence of PPGS. The surface of the pure PA56 fibres is relatively smooth with no obvious particles and sulphur EDS signals. In contrast, GS-2.0 exhibits a biphasic structure with aggregated PPGS particles of varying sizes dispersed in the PA56 matrix (Figure 4b), which can be ascribed to the large difference in polarity between PPGS and PA56. In the EDS images, clear and bright sulphur aggregates are observed. However, these aggregates disappeared after EMA addition (Figure 4c), which can be ascribed to the chemical reaction between the epoxy and amine groups during melt blending. EMA can also act as a compatibiliser and increase pliability by reducing interfacial tension and preventing particle agglomeration [39,40].

### 3.3. Effect of PPGS on the Thermal Stability of PA56

**Figure 5:**
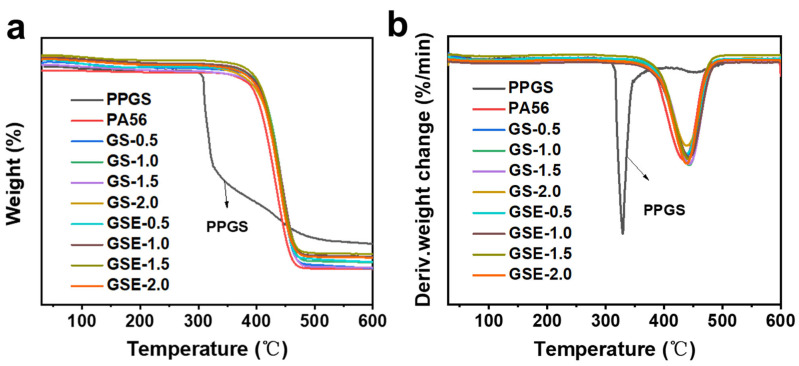
(**a**) TGA curves and (**b**) their derivatives for the samples heated under a nitrogen atmosphere.

TGA was used to investigate the effects of PPGS and EMA on the thermal stability of PA56. The weight and derivative weight change curves, as shown in Figure 5, show the similar trends of modified PA56 samples. The thermal parameters extracted from Figure 5 are shown in Table 2. Compared to PA56 with an onset decomposition temperature (T_5%_) of 381.5 °C, the T_5%_ of the modified samples decreases with increasing PPGS content, which can be ascribed to the lower decomposition temperature of PPGS than PA56. The T_5%_ of the samples with EMA are 5–10 °C higher than those of the PPGS blend samples, which may be attributed to the chemical bonding effects during processing. The typical processing temperature of PA56 is approximately 280 °C; thus, the addition of small amounts of PPGS is not expected to significantly affect its processability.

### 3.4. Mechanical Properties

**Figure 6:**
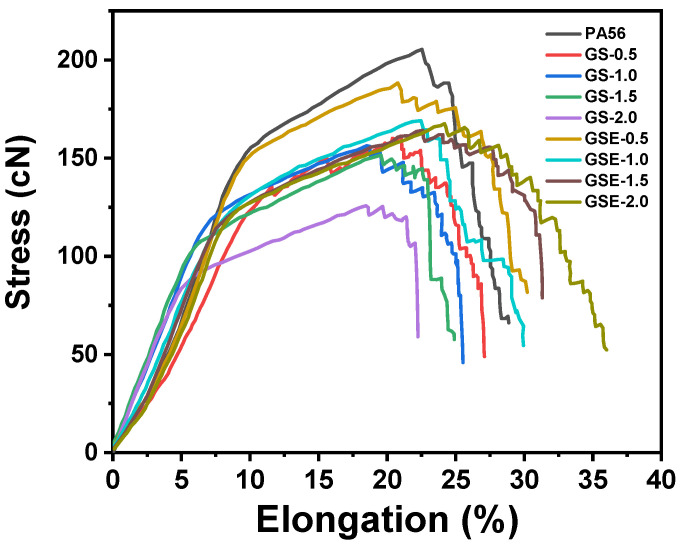
Stress–strain curves of PA56 modified fibers.

The mechanical properties of the fibres, which are crucial for practical applications, were evaluated. Figure 6 shows the tensile stress–strain curves for PA56 fibres 25 °C. All samples show the multiple yielding behavior. The mechanical properties of the fibres are summarized in Table 3. The elongation at break, tensile strength, and elastic modulus of the modified PA56 fibres continuously decrease with increasing PPGS addition (Table 3). Such decreases are ascribed to the poor compatibility between PPGS and PA56, resulting in an uneven stress distribution (i.e., stress concentration points), thereby weakening the fibres.

The elongation at break of the PA56 DTY fibres increased continuously with increasing EMA content, compared with PA56 fibres, the elongation at break of GSE-2.0 was increased by 25.93%. The epoxy groups in the molecular chains of the GSE fibres can react with the amine groups, increasing the compatibility between PA56, EMA, and PPGS. In contrast, EMA toughening agents can help form crazes and shear bands in the material [39,41,42]. Compared with fibres containing only PPGS, EMA toughening agents improve the compatibility of the blend samples, effectively absorbing stress and reducing stress concentration points inside the material, thereby enhancing the mechanical properties of the material.

### 3.5. Colorimetric Results

**Figure 7:**
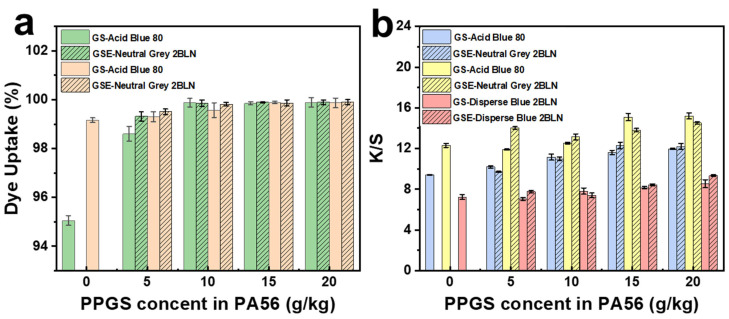
(**a**) Dye uptake by the fibres and (**b**) K/S values of dyed DTYs.

The dye uptake and K/S values of the dyed samples are shown in Figure 7. PA fibres are conventionally dyed with acid dyes [9,43], which easily combine with the amine groups of PA.

The cationic guanidine groups in PPGS bond with the dye molecules and improve the uptake of the acidic dyes by the modified fibres (Figure 7a) [44]. The colour strength of the fibres was quantified in terms of the K/S values, which increased significantly with increasing PPGS content in the fibres (Figure 7b), indicating darker fibres with improved dyeing performance.

Dispersed dyes are extensively applied to PA textiles, which can diffuse in the noncrystalline regions of the fibres and interact mainly through van der Waals forces, especially hydrogen bonds [45,46]. The UV absorbance of the Disperse Blue 2BLN solution was difficult to measure because of the nonsolubility of the dye in water. The dye uptake also can be analysed based on K/S values, whereby the dye uptake and K/S value are positively correlated. As shown in Figure 5b, the PPGS and EMA addition slightly increased the K/S values of the samples, which can be ascribed to the addition of some polar groups that contribute to fibre and dispersed dye binding [47].

**Figure 8:**
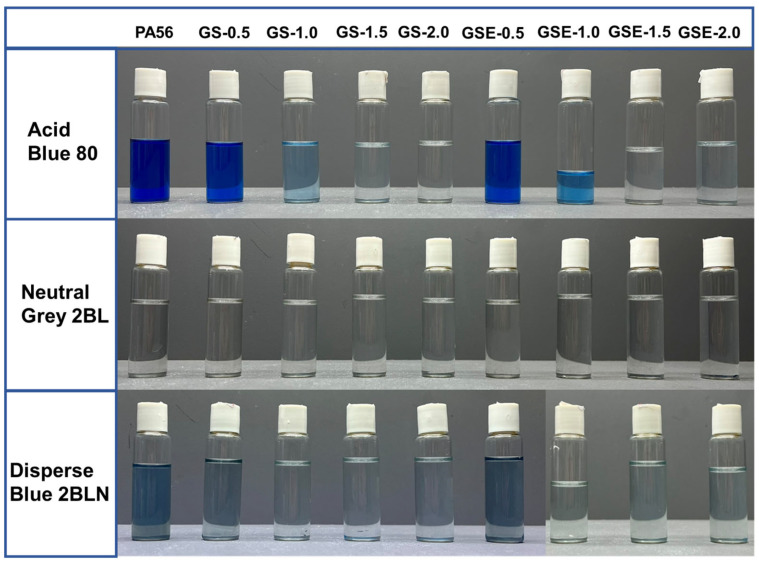
Residual liquids after washing the dyed fibres with soap solution.

To determine the effects of the PPMG content on the wash fastness of the dyes, washing tests were conducted according to ISO 105-C10 (2006) standard. Figure 8 shows photographs of the residual liquid after washing the dyed samples with a soap solution. The colour intensity of the residual liquid from washing the fibres dyed with Acid Blue 80 decreases as the PPMG content increases from 0.5 to 2.0 wt%, indicating an increase in colour fastness. The abundant amino groups provided by PPMG are considered to contribute to the wash fastness. In contrast, for the fibres dyed with Neutral Grey 2BL and Disperse Blue 2BLN, PPMG has minimal effects on the wash fastness. The wash fastness values for all samples are above level 4, which meets the requirements for industrial production.

### 3.6. Antibacterial Performance

**Figure 9:**
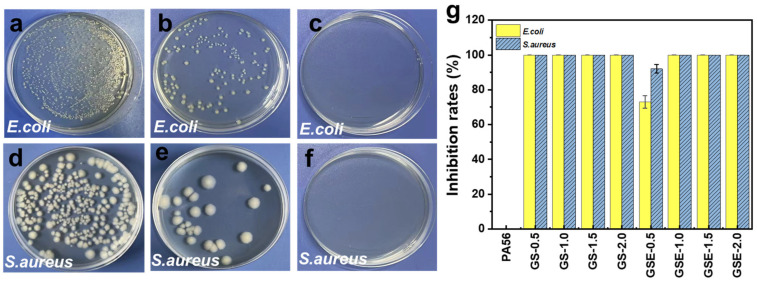
Typical antibacterial photographs of (**a**,**d**) PA56, (**b**,**e**) GSE-0.5, and (**c**,**f**) GS-0.5. (**g**) Inhibition rates of the fibres against *E. coli* and *S. aureus*.

The influence of PPGS incorporation on the antibacterial performance of the PA56 fibres was evaluated using the bacteria-counting method. Untreated PA56 fibres were used as the control samples (Figure 9a,d). After adding a small amount of PPGS, the fibres exhibit excellent antimicrobial activity, regardless of the EMA addition. With PPGS contents of more than 1.0 wt%, the bacterial inhibition rates exceed 99.99% (Figure 9g). GS-0.5 exhibits better antibacterial inhibition than GSE-0.5, (Figure 9b,c,e,f), which can be attributed to the improved compatibility between PPGS and PA56 and the epoxy-opening reaction that facilitated PPGS grafting onto PA56 by EMA, thereby minimizing the dissolution of PPGS. These findings are consistent with the SEM results. Additionally, the value of HC_50_ (64–128 ppm) indicated that PPGS had certain physiological toxicity (Figure S2). Therefore, the addition of EMA, which reduced the leaching of antibacterial agents, can retard the toxicity of antibacterial agents. 

The *E. coli* inhibition effects of the modified fibres before and after dyeing are presented in Table 4. The inhibition effect of the blended samples significantly decreases after dye-free processing with the inhibition rates of GS-0.5 and GS-1.0 dropping from 99.99% to 55.43% and 58.70%, respectively. This decrease is ascribed to the dissolution of PPGS under a long-term high-temperature dyeing process because the dye-free processing immerses the samples in water, with increasing temperature of the dye bath from room temperature to 98 °C at a heating rate of 1 °C/min and maintained at 98 °C for 30 min. However, the incorporation of EMA mitigated this trend. In particular, the inhibition rate of GSE-0.5 decreases by only 4% from 73.62% to 69.56% after dye-free processing. When the PPGS addition amount reaches 1.0 wt%, the antibacterial effect of the GSE series samples against *E. coli* is unchanged, indicating that the copolymerised samples achieved enhanced washability, which is favourable for practical use in fabrics. After dyeing with Acid Blue 80, the bacterial inhibition effect of the samples greatly decreases. Similarly, after dyeing with Disperse Blue 2 BLN, the bacterial inhibition effects of the fibres almost disappeared. The inhibition rates of GS-0.5 against *E*. *coli* decreases from 99.99% to 28.50% and 25.36% after dyeing with Acid Blue 80 and Dispersible Blue 2BLN, respectively. However, after dyeing with Neutral Grey 2BL, the antibacterial activities of the fibres containing EMA are maintained. Therefore, EMA addition in PA modification and the use of neutral dyes are important factors for ensuring the excellent antimicrobial activities of the modified fibres. The changes in the antibacterial properties of the fibres after dyeing were investigated.

### 3.7. Mechanism of PPGS and Dyes

**Figure 10:**
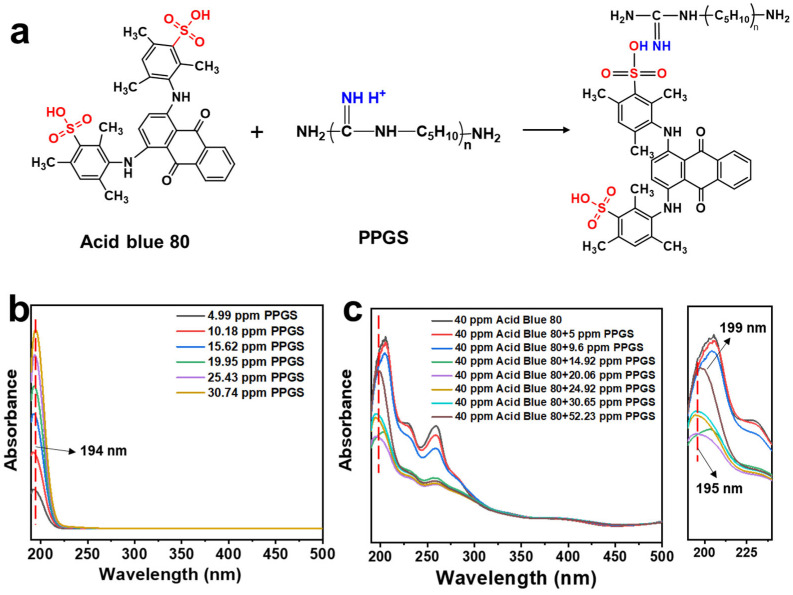
(**a**) Proposed reaction mechanism between Acid Blue 80 and PPGS. UV-vis spectra of solutions of (**b**) PPGS and (**c**) PPGS and Acid Blue 80.

The Acid Blue 80 molecule contains two –SO_3_Na groups that can form strong ion-ion bonding with the cationic guanidine groups of PPGS, reducing the dissociation of PPGS and consequently, the antibacterial efficacy, which is consistent with previous studies [48,49,50]. An electrostatic interaction mechanism between Acid Blue 80 and PPGS is proposed (Figure 10a), which was verified using UV-visible spectroscopy. A series of PPGS aqueous solutions were prepared, and their absorption spectra were obtained in the range of 190–500 nm by UV absorption spectroscopy (Figure 10b). With increasing PPGS concentration, the intensity of the absorption peak of the guanine groups in the solution (194 nm) increases. Figure 10c shows that the spectra of the 40 ppm Acid Blue 80 dye solution. No significant absorption peaks at 194 nm are noted for the samples with less than 15 ppm PPGS. Meanwhile, the absorption peak of the Acid Blue 80 dye gradually decreases with increasing PPGS content. When the PPGS concentration exceeded 20 ppm, a distinct guanidine absorption peak is observed at 195 nm, which redshifted from 194 to 199 nm as the PPGS concentration was increased. This can be ascribed to the interaction between the guanidine and sulphonic acid groups, which enlarges the conjugated structure, resulting in reduced PPGS dissociation.

**Figure 11:**
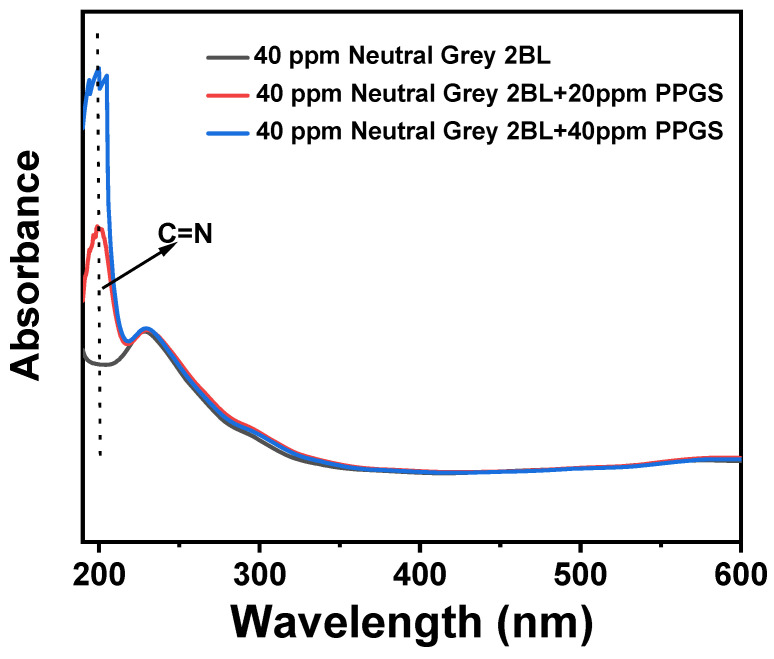
UV–vis spectra of the solutions of PPGS and Neutral Grey 2BL.

Neutral dye molecules are electrically neutral, i.e., they do not ionise in solution. Figure 11 shows the spectra of the solutions of PPGS and Neutral Grey 2BL. No significant changes in the intensity and location of the absorption peak are observed. The increased absorption peak at approximately 195 nm is ascribed to the increased PPGS concentration. Therefore, dyeing with Neutral Grey dye is expected to have minimal effects on the antibacterial effect of the fibres.

**Figure 12:**
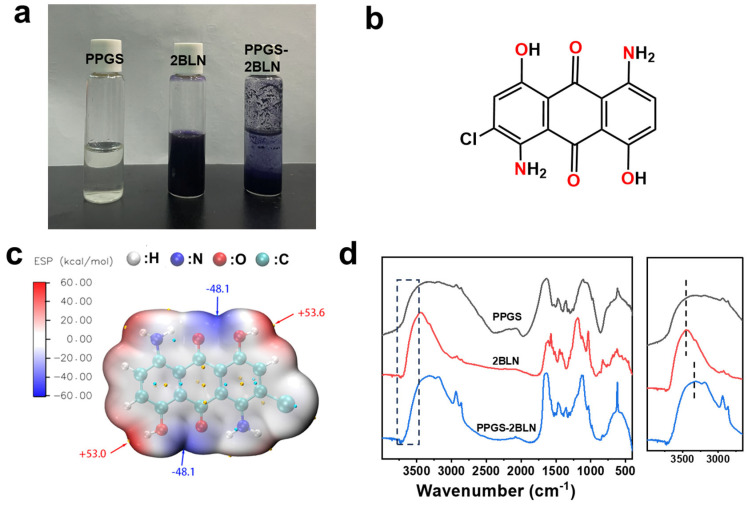
(**a**) Photograph of PPGS and Disperse Blue 2BLN solutions. (**b**) Chemical structure and (**c**) molecular electrostatic potential map of Disperse Blue 2BLN. (**d**) FTIR spectra of PPGS and Disperse Blue 2BLN.

When Disperse Blue 2BLN dye was added to the PPGS aqueous solution, immediate precipitation is observed (Figure 12a). The chemical structure of Disperse Blue 2BLN is shown in Figure 12b. To further investigate the properties of Disperse Blue 2BLN, its molecular conformation was optimised using Gaussian 09 based on the B3LYP/6-31G theory. The calculation of the electrostatic potential at the van der Waals surface using the Multiwfn Software is shown in Figure 12c. The Disperse Blue 2BLN molecule has polar groups (–NH_2_, –OH, etc.) with significant electropositivity (+53.6 kJ/mol) and electronegativity (−48.1 kJ/mol). Areas with large electrical potentials tend to form electrostatic interactions or hydrogen bonds [45,46]; this theory supports the IR spectra (Figure 12d), which exhibits broadening of the peaks related to –OH and –NH groups and a blue shift. The interaction between PPGS and Disperse Blue 2BLN may be a significant factor of the decline in the antibacterial function of the modified fibres.

## 4. Conclusions

PPGS was successfully introduced into PA56 fibres via melt blending. The influence of PPGS, which acted as an antimicrobial agent, and EMA, which acted as a compatible coupling agent, on the performance of PA56 DTY was systematically investigated. The chemical bonding analysis of PPGS in the fibres revealed that the addition of EMA significantly improved the compatibility between PPGS and PA, and improved the mechanical properties of the fibre modified by the blend system. The antimicrobial activities of PA56 DTYs were significantly improved by PPGS incorporation, with inhibition rates of 99.99% against *E. coli* and *S. aureus*. The colour strength of the fibres dyed with three different representative dyes increased slightly after modification. The washing fastness values of the various DTY samples were above Level 4, confirming that the stability of the dye on the fibres was not negatively affected by the addition of PPGS and EMA, above all, the dissolution of PPGS was reduced after the addition of EMA. Nonetheless, the antimicrobial efficiencies of DTYs dyed with Acid Blue 80 and Disperse Blue 2BLN were significantly lower after PPGS and EMA addition. Therefore, neutral dyes are the most suitable choice for dyeing guanidine-salt-modified PA56 fibres. This study provides a practical and accessible method for preparing antibacterial PA56 textiles, which is expected to expand the application potential of PA-based materials.

## Figures and Tables

**Table 1:** Compositions of the samples.

Sample Name	PPGS Content (wt%)	EMA Content (wt%)	Bio-Based PA56 Content (wt%)
PA56	0.0	0.0	100.0
GS-0.5	0.5	0.0	99.5
GS-1.0	1.0	0.0	99.0
GS-1.5	1.5	0.0	98.5
GS-2.0	2.0	0.0	98.0
GS-10.0*	5.0	0.0	95.0
GSE-0.5	0.5	0.5	99.0
GSE-1.0	1.0	1.0	98.0
GSE-1.5	1.5	1.5	97.0
GSE-2.0	2.0	2.0	96.0
GSE-10.0 *	5.0	5.0	90.0

* Reference chips.

**Table 2:** Thermal parameters of the samples by heading obtained under a nitrogen atmosphere.

Sample	T_5%_ (°C)	T_max_ (°C)
PPGS	310.8	328.3
PA56	381.5	432.4
GS-0.5	368.5	438.2
GS-1.0	363.9	443.0
GS-1.5	359.2	443.1
GS-2.0	347.6	438.1
GSE-0.5	373.6	438.3
GSE-1.0	370.9	443.1
GSE-1.5	367.4	440.9
GSE-2.0	363.2	438.6

**Table 3:** Mechanical properties of the PA56 DTY fibres.

Samples	Elongation at Break (%)	Breaking Strength (cN/dtex)	Elastic Modulus (cN/dtex)
PA56	27.27 ± 0.94	4.11 ± 0.33	38.11 ± 2.65
GS-0.5	26.08 ± 0.21	3.91 ± 0.37	32.28 ± 1.63
GS-1.0	22.51 ± 1.97	3.83 ± 0.42	31.05 ± 1.73
GS-1.5	20.24 ± 1.17	3.27 ± 0.22	25.67 ± 0.85
GS-2.0	17.01 ± 1.28	2.89 ± 0.44	20.65 ± 1.23
GSE-0.5	27.75 ± 1.45	3.93 ± 0.35	33.69 ± 1.37
GSE-1.0	28.29 ± 1.57	3.86 ± 0.33	32.86 ± 0.56
GSE-1.5	30.85 ± 1.20	3.62 ± 0.17	26.56 ± 0.72
GSE-2.0	34.85 ± 1.29	3.34 ± 0.42	24.44 ± 1.36

**Table 4:** Inhibition rates (%) against *E. coli* measured using the shaking flask method.

Samples	Before Dyeing	After Dye-Free Processing	After Acid Blue 80 Dyeing	After Neutral Grey 2BL Dyeing	After Disperse Blue 2BLN Dyeing
GS-0.5	>99.99	55.43	28.50	53.25	25.36
GS-1.0	>99.99	58.70	39.12	55.82	22.43
GS-1.5	>99.99	88.04	45.58	85.64	20.68
GS-2.0	>99.99	97.83	59.19	95.23	34.24
GSE-0.5	73.62	69.56	56.02	68.53	27.92
GSE-1.0	>99.99	>99.99	82.57	>99.99	28.71
GSE-1.5	>99.99	>99.99	85.31	>99.99	36.14
GSE-2.0	>99.99	>99.99	87.90	>99.99	57.77

## Data Availability

The original contributions presented in the study are included in the article/Supplementary Material, further inquiries can be directed to the corresponding author.

## Supplementary Materials

The following supporting information can be downloaded at: https://www.mdpi.com/article/10.3390/polym16192707/s1, Section S1: Experiments; Section S2: Characterization of Haemolytic Assay; Section S3: Results of RBC Haemolysis; Figure S1: Synthesis of bio-based PPMG; Figure S2: RBC haemolysis for PPGS; Table S1: Chemical structures of the dyes.
