# Supplementary material for: Guanidine Derivatives Leverage the Antibacterial Performance of Bio-Based Polyamide PA56 Fibres
Source: Polymers (Basel). 2024 Sep 25;16(19):2707. doi: 10.3390/polym16192707 (PMC11478546; doi:10.3390/polym16192707)
Supplement: Supplementary file 1 [file polymers-16-02707-s001.zip › polymers-3181762-supplementary.pdf]

Supporting Information

Guanidine Derivatives Leverage the Antibacterial Performance of Bio-based Polyamide PA56 fibres

Lili Wang[1], Bobo Zhou[1], Yuliu Du[2], Miao Bai[2], Xiang Xu[1], Yong Guan[1,], and Xiucai Liu[1,2,*]*

[1]*School of Materials Science and Engineering, East China University of Science and Technology, Shanghai 200237, China*

[2] *Shanghai Cathay Biotechnology Co., Ltd. Shanghai, 201144, China*

*Corresponding Author. E-mail: xiucliu@cathaybiotech.com (X. Liu), yguan@ecust.edu.cn (Y. Guan)

Contents

S1. Experiments 2
S1.1 Synthesis of antimicrobial agent. 2
S1.2 Preparation of the functional antibacterial bio-based PA56. 2
S1.3 Chemical structures of the studied dyes 3
S2. Characterisation of Haemolytic Assay 3
S3. Results of RBC Haemolysis 4
Reference 4

S1. Experiments

S1.1 Synthesis of antimicrobial agent. Polypentamethylene guanidine hydrochloride (PPMG) was synthesised according to the procedure reported in the literature.[1] The synthesis scheme of bio-based PPMG is shown in Figure S1. Polycondensation bio-based PDA and guanidine hydrochloride with equimolar ratio was firstly carried in a glass reactor at 120 °C for 2 h and then increased to 200 °C for 6 h. Ammonia, as a byproduct of the reaction, was absorbed by the HCl aqueous solution. At the end of the polymerisation, a light-yellow viscous product was then vacuum dried at 80 °C for 48 h to remove residual ammonia.

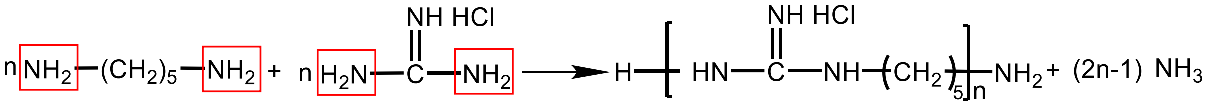

Figure S1. Synthesis of bio-based PPMG.

Subsequently, PPMG was dissolved in deionised water to form a 15 wt% solution, and ion exchange was used to remove Cl^- from the PPMG solution. The solution of filtered effluent was obtained prior to its saturation, and excess water was removed using a rotary evaporator to obtain a pure antibacterial agent, which was recorded as PPGS.

S1.2 Preparation of the functional antibacterial bio-based PA56. Before preparing the blends, PA56 was dried in a vacuum oven at 105 °C for 8 h. EMA was dried in the vacuum oven at 50 °C for 24 h. Different ratios of PA56, PPMG, and EMA were mixed and added into a TDS-26B (D=26mm and L/D=40) co-rotating twin extruder (Nanjing Norda Extrusion Equipment Co., Ltd. China) under the following temperature profiles: 170, 250, 260, 270, 270, 270, 270, and 275 °C; the screw rotation speed was set at 350 rpm with a controlled feed rate of 35rpm. The extruded strand was collected through a water bath, granulated, and dried at 105°C for 6 h before further processing. The proportions and definitions of the samples are shown in Table S1. For comparative purposes, all samples were

processed and dried under the same conditions.

Before the preparation of fibre samples, the chips were dried in a vacuum oven at 105 °C for 8 h to keep the moisture content of the dried chips at approximately 400 ppm. The spinning process was as follows: The chips were melted and metered using a screw extruder and the melt was used to prepare fully drawn filament POY samples. The temperature of each zone of the screw was 250 °C, 260 °C, 285 °C, 285 °C, 285 °C, and 285 °C. Other parameters include cooling by side air with an air temperature of 17 °C, a spinning temperature of 285 °C, a spinning speed of 4200 m/s, a cooling blowing speed of 0.40 m/s, and a draw ratio of 1.25, with the temperature of the first heating roller being 70°C, the temperature of the second heating roller being 150°C, and an oil content 1.2%.

S1.3 Chemical structures of the studied dyes

Table S1. Chemical structures of the dyes.

Dye No.1: Acid Blue 80	Dye No.2: Neutral Grey 2BL	Dye No.3: Disperse Blue 2BLN
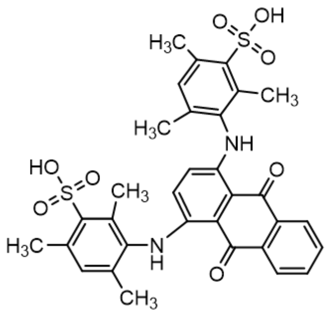	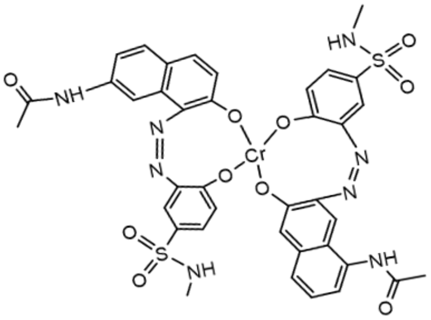	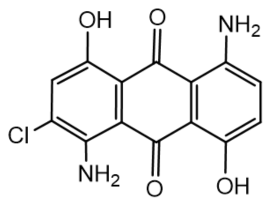

S2. Characterisation of Haemolytic Assay

Fresh sheep blood was washed with Tris-buffered saline (TBS) three times and the obtained sheep red blood cells (SRBCs) were diluted to 5% (v/v) with TBS. The PPGS was diluted to concentrations ranging from 1 to 512 ppm by a two-fold gradient dilution in a 96-well plate. After

mixing an equal volume of SRBC suspension and PPGS solution, 96-well plates were incubated at 37 °C for 1 h. TBS was used as the blank; the mixture of Triton X-100 (3.2 ppm in TBS) and SRBC

was used as the positive control. After centrifugation, 80 μL of the supernatant in each well was transferred to another 96-well plate and the OD value was collected to calculate the percentage of haemolysis using Equation S1 below; the results were calculated based on the average of three repeat tests.

$$\% \; hemolysis = \frac{A_{405}^{PPGS} - A_{405}^{blank}}{A_{405}^{control} - A_{405}^{blank}} \times 100 \qquad \text{S1}$$

S3. Results of RBC Haemolysis

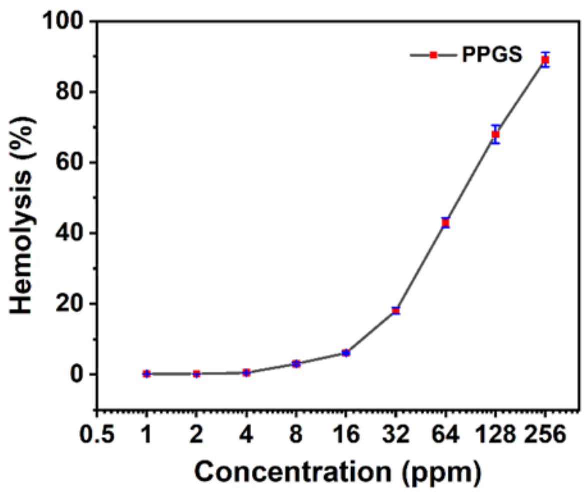

Figure S2. RBC haemolysis for PPGS.

The value of HC_{50} for PPGS is 64–128 ppm, which indicated that PPGS had a certain physiological toxicity. Therefore, in practical application, PPGS is recommended to be combined with materials via chemical bonding to prepare non-dissolved antibacterial materials.

Reference

(1) Shentu, X.; Guan, Y.; Wang, L.; Pan, J.; Zheng, A.; Wei, D.; Xu, X. Preparation of antibacterial down fibers by chemical grafting using novel guanidine salt oligomer. *Polymers for Advanced Technologies* **2021**, *32*, 4082-4093.

(2) Wang, Y.; Kang, H.; Guo, Y.; Liu, R.; Hao, X.; Qiao, R.-r.; Yan, J.-l. The structures and properties of bio-based polyamide 56 fibers prepared by high-speed spinning. *Journal of Applied Polymer Science* **2020**, *137* (44), 49344.

(3) Gottlieb, H. E. K., Vadim; Nudelman, Abraham. NMR Chemical Shifts of Common Laboratory

Solvents as Trace Impurities. *J. Org. Chem.* **1997**, *62*, 7512-7515. DOI: 10.1021/jo971176v.
